# Bacteriome and mycobiome associations in oral tongue cancer

**DOI:** 10.18632/oncotarget.21921

**Published:** 2017-10-19

**Authors:** Pranab K. Mukherjee, Hannah Wang, Mauricio Retuerto, Huan Zhang, Brian Burkey, Mahmoud A. Ghannoum, Charis Eng

**Affiliations:** ^1^ Center for Medical Mycology, Department of Dermatology, School of Medicine, Case Western Reserve University, Cleveland, OH, USA; ^2^ Genomic Medicine Institute, Lerner Research Institute, Cleveland Clinic, Cleveland, OH, USA; ^3^ Cleveland Clinic Lerner College of Medicine of Case Western Reserve University, Cleveland, OH, USA; ^4^ Taussig Cancer Institute, Cleveland Clinic, Cleveland, OH, USA; ^5^ Section of Head and Neck Surgery and Oncology, Head and Neck Institute, Cleveland Clinic, Cleveland, OH, USA; ^6^ Department of Genetics and Genome Sciences, Case Western Reserve University School of Medicine, Cleveland, OH, USA; ^7^ Department of Dermatology, University Hospitals Cleveland Medical Center, Cleveland, OH, USA; ^8^ Germline High Risk Cancer Focus Group, CASE Comprehensive Cancer Center, Case Western Reserve University School of Medicine, Cleveland, OH, USA

**Keywords:** microbiome, metagenomics, head and neck squamous cell carcinoma, lingual carcinomas

## Abstract

Squamous cell carcinoma of the oral (mobile) tongue (OMTC), a non-human papilloma virus-associated oral cancer, is rapidly increasing without clear etiology. Poor oral hygiene has been associated with oral cancers, suggesting that oral bacteriome (bacterial community) and mycobiome (fungal community) could play a role. While the bacteriome is increasingly recognized as an active participant in health, the role of the mycobiome has not been studied in OMTC. Tissue DNA was extracted from 39 paired tumor and adjacent normal tissues from patients with OMTC. Microbiome profiling, principal coordinate, and dissimilarity index analyses showed bacterial diversity and richness, and fungal richness, were significantly reduced in tumor tissue (TT) compared to their matched non-tumor tissues (NTT, *P*<0.006). Firmicutes was the most abundant bacterial phylum, which was significantly increased in TT compared to NTT (48% vs. 40%, respectively; *P*=0.004). Abundance of Bacteroidetes and Fusobacteria were significantly decreased in TT compared to matched NTT (*P*≤0.003 for both). Abundance of 22 bacterial and 7 fungal genera was significantly different between the TT and NTT, including *Streptococcus*, which was the most abundant and significantly increased in the tumor group (34% vs. 22%, *P*<0.001). Abundance of fungal genus *Aspergillus* in TT correlated negatively with bacteria (*Actinomyces, Prevotella*, *Streptococcus)*, but positively with *Aggregatibacter*. Patients with high T-stage disease had lower mean differences between TT and NTT compared with patients with low T-stage disease (0.07 vs. 0.21, *P*=0.04). Our results demonstrate differences in bacteriome and mycobiome between OMTC and their matched normal oral epithelium, and their association with T-stage.

## INTRODUCTION

Squamous cell carcinoma of the oral (mobile) tongue, arising at the anterior two-thirds of the tongue, has been rapidly increasing and has now become the second most common malignancy in the oral cavity [1, 2]. While human papillomavirus (HPV) is etiologic for the increased incidence of base of tongue tumors (89% HPV+), HPV is rarely found (2.3%) in oral tongue cancer [3-5]. The etiology of this increasingly common disease has remained unclear, and warrants investigation for discovery of additional pathogenic pathways.

The microbiome, defined as the total collection of microorganisms that inhabit any environment, is increasingly recognized as an active participant in human body functions, and has been even proposed to be an organ. The microbiota comprise bacterial community (bacteriome) and fungal community (mycobiome).

In/on the human body, bacterial cells outnumber human cells 10:1, with the total bacteriome-to-human gene content ratio approximating 350:1. The notion that bacteria can be oncogenic is demonstrated in the example of *Helicobacter pylori* in gastric cancer [6], and more recently, *Fusobacterium nucleatum* in colorectal cancer [7, 8]. The discovery of *H. pylori* as a trigger for gastric cancer shifted the paradigm of oncogenesis to one that includes bacteria. Studying the microbiota was largely limited to culturable organisms before the advent of metagenomics, the detection of the genomic content of microbes. Metagenomics now allows for detection of both culturable and nonculturable microbes, permitting us to describe bacterial communities.

Our pilot work profiling the microbiome of 42 mixed (heterogenous) head and neck squamous cell carcinomas (HNSCCs) revealed that microbial variation could correlate with clinical outcomes such as nodal and tumor stage as well as gene methylation status, but was limited by diversity of involved sites [9]. Another study with a more limited sample size used superficial bacterial sampling of 15 oral cavity cancers by means of oral swabs, and found differences in taxonomic abundance between normal and tumor surfaces at the phylum level [10]. However, bacteria in the head and neck populate both the deep and mucosal tissues [9, 11, 12]. With increasing evidence that a rich community of bacteria live within the oral cavity, it follows that bacterial inhabitants should contribute to the tumor microenvironment.

In addition to the bacteriome, another major component of the human microbiome is the mycobiome. Recent studies point to the importance of our commensal fungal inhabitants as critical players in human health and disease [13]. Although the role of the mycobiome in oral cancer, including oral tongue cancer, has not been investigated, a recent study by Shelburne *et al.* [14] combined genetic analyses of the infecting agent, host whole exome sequencing, and longitudinal determination of the oral and stool micro- and mycobiomes in a leukemic patient, and suggested that the dysbiotic nature of the oral bacteriome may have provided a permissive environment for establishment and the eventual development of invasive mucormycosis. Their findings generated new interest in understanding mechanisms driving maintenance or loss of microbial diversity during cancer therapy. Moreover, studies are starting to emerge demonstrating interactions between bacteria and fungi (i.e. inter-kingdom interactions) suggesting that bacteriome or mycobiome alone may not exclusively play a role in disease pathogenesis [15, 16]. Thus, studies aimed at understanding how these two communities influence or are influenced in disease setting such as oral tongue cancer are needed.

Based on the above, we sought to explore the bacteriome and mycobiome in mobile tongue cancers. In this exploratory study, we selected oral tongue cancers to minimize confounding contributions from HPV and from multiple oral sites, and sought to determine whether there were differences in the bacteriome and/or mycobiome between oral tongue cancers and matched normal tissue, and to evaluate if bacteriome and/or mycobiome differences are correlated with clinico-pathologic features.

## RESULTS

**Figure 1:**
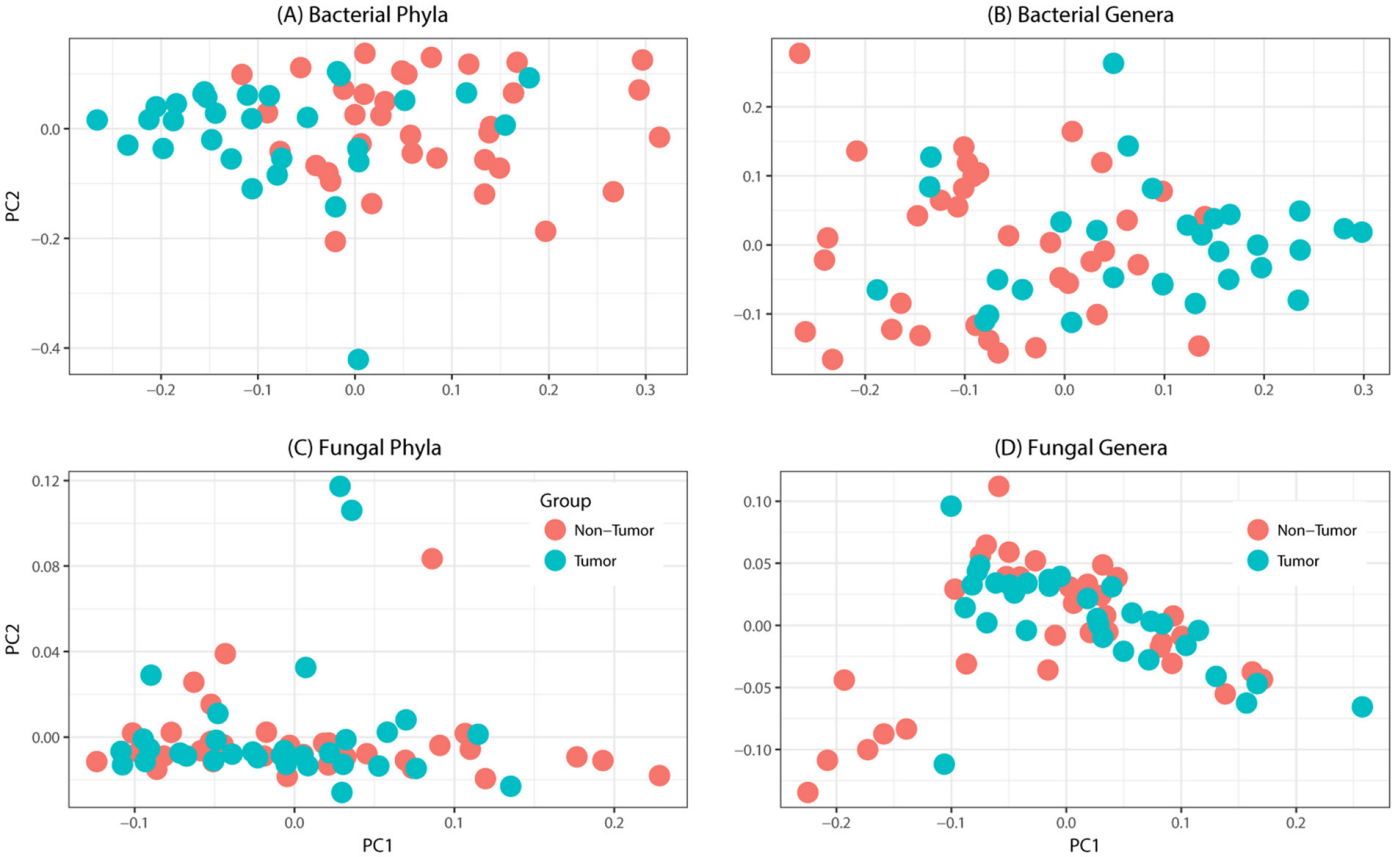
Principal coordinates analysis of oral tongue tumor samples and matched non-tumor oral epithelium samples at the phylum (left) and genus (right) levels for **(A-B)** bacteriome or **(C-D)** mycobiome. Overall oral microbiomic diversity of patient tumor (green) and matched non-tumor (orange) samples as represented by the first two principal coordinates on principal coordinates analysis of unweighted UniFrac distances. Each point represents a single sample.

Tissue samples from 39 patients were analyzed in this study (Table 1). The total number of reads associated with bacteriome and mycobiome were 3,093,772 and 4,550,121 respectively, which were passed through quality filters, resulting in 1,997,240 and 1,149,569 sequences used for OTU assignments (26,279 and 14,369 mean sequences per sample, respectively). Non-tumor samples had significantly higher numbers of reads compared to tumor samples for both bacterial (median, Q1-Q3; 13,561, 8,522-18,804 vs. 6,294, 592-11,504; *P* < 0.0001) and fungal sequences (3,482, 1,493-6168 vs. 1,806, 654-3119; *P* = 0.005). Principal coordinate analysis (PCO), clustered dendograms, and Bray-Curtis dissimilarity index analyses showed that the tumor and non-tumor samples exhibited considerable overlap in clustering at both phylum and genus levels, for bacterial and fungal biota (Figure 1). A total of 7 fungal and 25 bacterial phyla were identified in the collected samples.

**Table 1:** Demographic and clinical characteristics of study patients

Variable	All Patients (N = 39)
Age (years)	60.5 ± 13.2
Male	30 (76.9)
Race	
White	32 (88.9)
Non-White	4 (11.1)
Unknown	3
T-stage	
Low T-stage (T1-T2)	17 (45.9)
High T-stage (T3-T4)	20 (54.1)
Unknown	2
N-stage	
Node Negative (N0)	19 (51.4)
Node Positive (N1-N2)	18 (48.6)
Unknown	2
Overall Stage	
Stage I-II	7 (20.6)
Stage III-IV	27 (79.4)
Unknown	5
Smoking History	
Current	7 (17.9)
Past	19 (48.7)
Never	13 (33.3)
Alcohol Use	
Heavy	2 (5.2)
Social	14 (36.8)
Histroy	6 (15.8)
Never	16 (42.1)
Unknown	1
Site	
CHTH	10 (25.6)
HN	27 (69.2)
Duke	1 (2.6)
Vanderbilt	1 (2.6)

Values are presented as means ± standard deviations or number (percent). Percentages were calculated out of a denominator that does not include samples with missing data.

**Figure 2:**
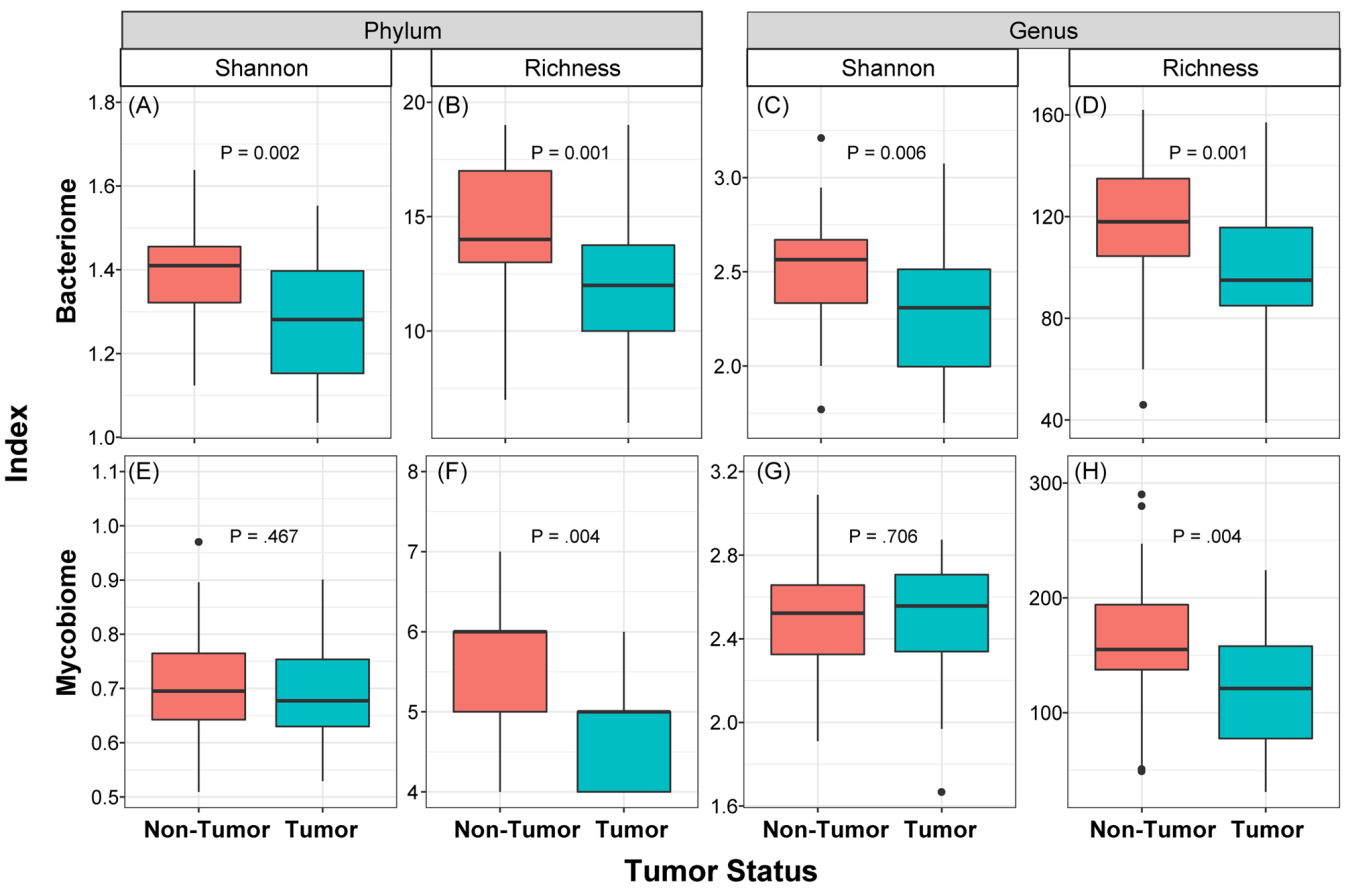
Box and whisker plots of diversity and richness of mycobiome and bacteriome at the phylum and genus levels from oral tongue tumor (green) and their matched non-tumor (orange) samples Diversity was analyzed in an unbiased manner using Shannon diversity index **(A, C, E, G)**, which characterizes species diversity, and richness **(B, D, F, H)** represents number of organisms in a given sample. Dark horizontal lines represent the median, with the box representing the first (Q1) and third (Q3) quartiles, the outer fences representing 1.5 x interquartile range, and the black circles representing outliers.

We determined the Shannon diversity index (measure of microbial abundance taking into account their distribution) as well as richness (species count, without taking into account the abundance) of bacterial and fungal communities in the collected samples. These analyses revealed that diversity and richness of the bacteriome, and richness of the mycobiome, in tumor samples were significantly reduced compared to their matched non-tumor samples (P≤0.006, Figure 2). No significant difference in Shannon diversity index was observed for the mycobiome between tumor and non-tumor samples at either phylum or genus levels (P>0.05, Figure 2E, 2G). Within the tumor group, there were no significant differences when comparing diversity indices by different gender (male vs. female), race, age (≤ 40 years vs. older), or smoking status (never smoked vs. current or past smokers, data not shown). Interestingly, richness of both bacterial and fungal phyla was significantly increased in high T-stage tumor samples compared to low T-stage samples (*P*≤0.047, Supplementary Figure 1). Within the non-tumor (matched normal) tissues, there was no difference in diversity indices between different T-stage, age or race. However, richness was significantly decreased among fungal phyla in smokers compared to non-smokers in normal non-tumor samples (4.96 ± 0.82 vs. 5.08 ± 0.79, *P*=0.045).

**Figure 3:**
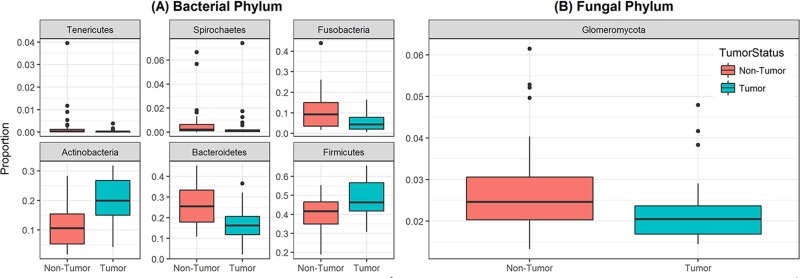
Box and whisker plots of relative abundance of the 6 bacterial phyla and one fungal phylum found significantly different between oral tongue tumor samples and their matched normal tissue samples **(A)** Box and whisker plots representing relative abundances of bacterial phyla Tenericutes, Spirochaetes, Fusobacteria, Actinobacteria, Bacteroidetes and Firmicutes by sample type non-tumor tissues (orange) and oral tongue tumors (green). **(B)** Box and whisker plots representing relative abundance of fungal phylum Glomeromycota in non-tumor tissues (orange) and oral tongue tumors (green). Dark horizontal lines represent the median, with the box representing the first (Q1) and third (Q3) quartiles, the outer fences representing 1.5 x interquartile range, and the black circles representing outliers. *P* <.05 for all comparisons between non-tumor and tumor groups.

Analysis of relative abundance at the phylum level revealed that 6 bacterial phyla and one fungal phylum were significantly different between non-tumor and tumor groups (Figure 3). Among the bacterial phyla, Firmicutes was the most abundant, and significantly increased in the tumor group compared to their matched non-tumor tissues (48% vs. 40%, respectively; *P*=0.004, Figure 3A). Levels of Actinobacteria were also significantly increased in tumor group compared to non-tumor tissue (20% vs. 11%, *P*<0.001). In contrast, abundance of Bacteroidetes and Fusobacteria was significantly decreased in the tumor group compared to matched non-tumor samples (P≤0.003 for both phyla). The fungal phylum Glomeromycota was significantly decreased in the tumor group compared to their matched non-tumor tissues (Figure 3B, 2.2% vs. 2.7%, *P*=0.01). There was no difference in abundance of any other fungal phyla between the tumors and their matched non-tumor tissues. Within the tumor group, there were no significant differences when comparing abundance levels between different gender (male vs. female), race, age (≤40 vs. older), or smoking status (data not shown). Interestingly, relative abundance of three bacterial phyla (Tenericutes, Spirochaetes, and Bacteroidetes) were significantly increased in high T-stage samples compared to low T-stage samples (*P*= 0.022, 0.009, and 0.046, respectively, Supplementary Figure 2). Within the non-tumor (matched normal) tissues, relative abundance of Spirochaetes increased in high T-stage samples compared to low T-stage samples (*P*=0.018), while that of Fusobacteria increased in smokers compared to non-smokers (*P*=0.02).

At the genus level, 22 bacterial and 7 fungal genera were significantly different in abundance in the tumor and non-tumor groups (Supplementary Tables 1, 2). Among the 22 significantly different bacterial genera, *Streptococcus* was the most abundant, and was significantly increased in the tumor group compared to their matched non-tumors (34% vs. 22%, *P*< 0.001). In addition, levels of *Actinomyces* and *Rothia* (and three lower abundance genera, *Corynebacterium*, *Enterococcus*, *Micrococcus*) were also significantly increased in the tumor group (*P*≤ 0.017, Supplementary Table 1). Fungal Genus *Wallemia* was the most abundant in both tumor and non-tumor groups (1.23% and 1.24%, *P*= 0.047, Supplementary Table 2). While six additional fungal genera differed significantly in abundance between the two groups (*P*≤ 0.043), their mean abundance was less than 1%. Among bacterial species, abundance of *Rothia mucilaginosa* was significantly increased in tumor samples compared to non-tumor samples (Table 2, 27% vs. 13%, *P*≤ 0.001).

**Table 2:** Abundance of bacterial species in non-tumor and oral tongue tumor tissues

Species	Non-Tumor		Tumor		_*P*_
	Mean	SD	Mean	SD	
*Rothia mucilaginosa*	13.43%	10.25%	27.78%	17.55%	<0.001
*Prevotella nigrescens*	8.66%	12.68%	4.93%	9.20%	0.027
*Porphyromonas endodontalis*	3.75%	9.07%	0.53%	0.43%	0.011
*Peptostreptococcus anaerobius*	3.09%	9.24%	1.14%	4.47%	0.006
*Capnocytophaga ochracea*	1.86%	5.43%	1.70%	7.14%	0.005
*Aggregatibacter segnis*	1.16%	3.06%	0.28%	0.28%	0.003
*Rothia dentocariosa*	0.72%	1.27%	1.48%	1.83%	0.003
*Prevotella copri*	0.45%	0.36%	0.56%	1.51%	0.039
*Sphingobacterium multivorum*	0.16%	0.73%	0.01%	0.02%	0.010
*Haemophilus influenzae*	0.13%	0.33%	0.05%	0.19%	0.001

**Figure 4:**
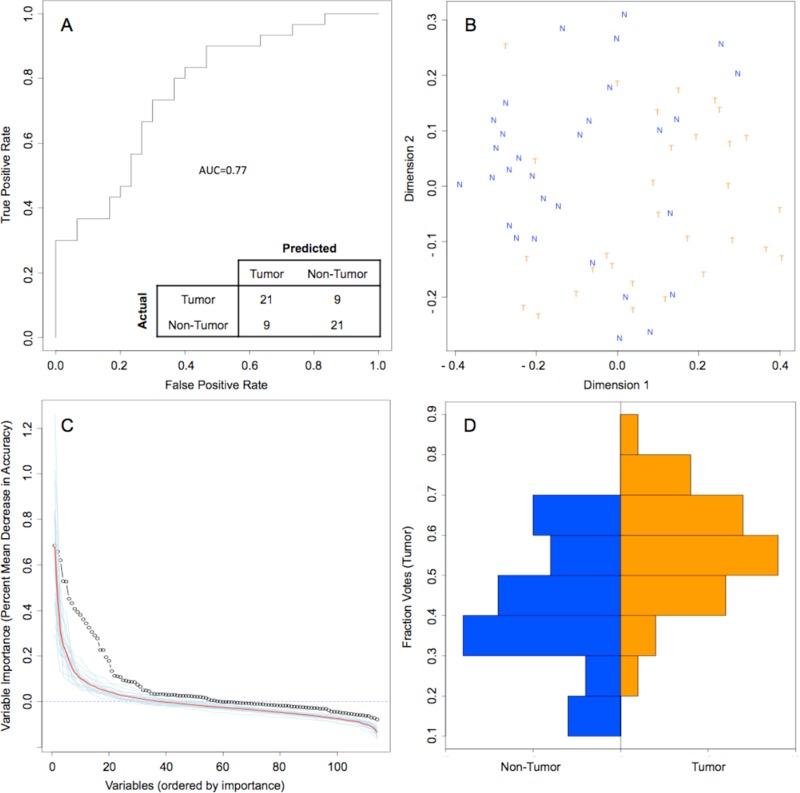
Random Forest model of genus-level bacteriome and mycobiome of oral tongue tumors compared to matched non-tumor tissues **(A)** ROC curve for model, with a bootstrapped estimated out-of-bag error rate of 0.30. **(B)** Multidimensional scaling plot of proximity matrix with non-tumor samples (blue “N”) clustering at the top left and tumor samples (orange “T”) clustering at the bottom right. **(C)** Screen plot of variable importances (black circle) for this model as compared to 20 models generated on random groupings (individual random models = blue lines, average of random models = red line). **(D)** Histograms of vote distributions for non-tumor (blue, left), and tumor (orange, right) samples. Vote fractions above 0.5 indicate samples predicted as tumor, while vote fractions below 0.5 indicate samples predicted as non-tumor.

**Figure 5:**
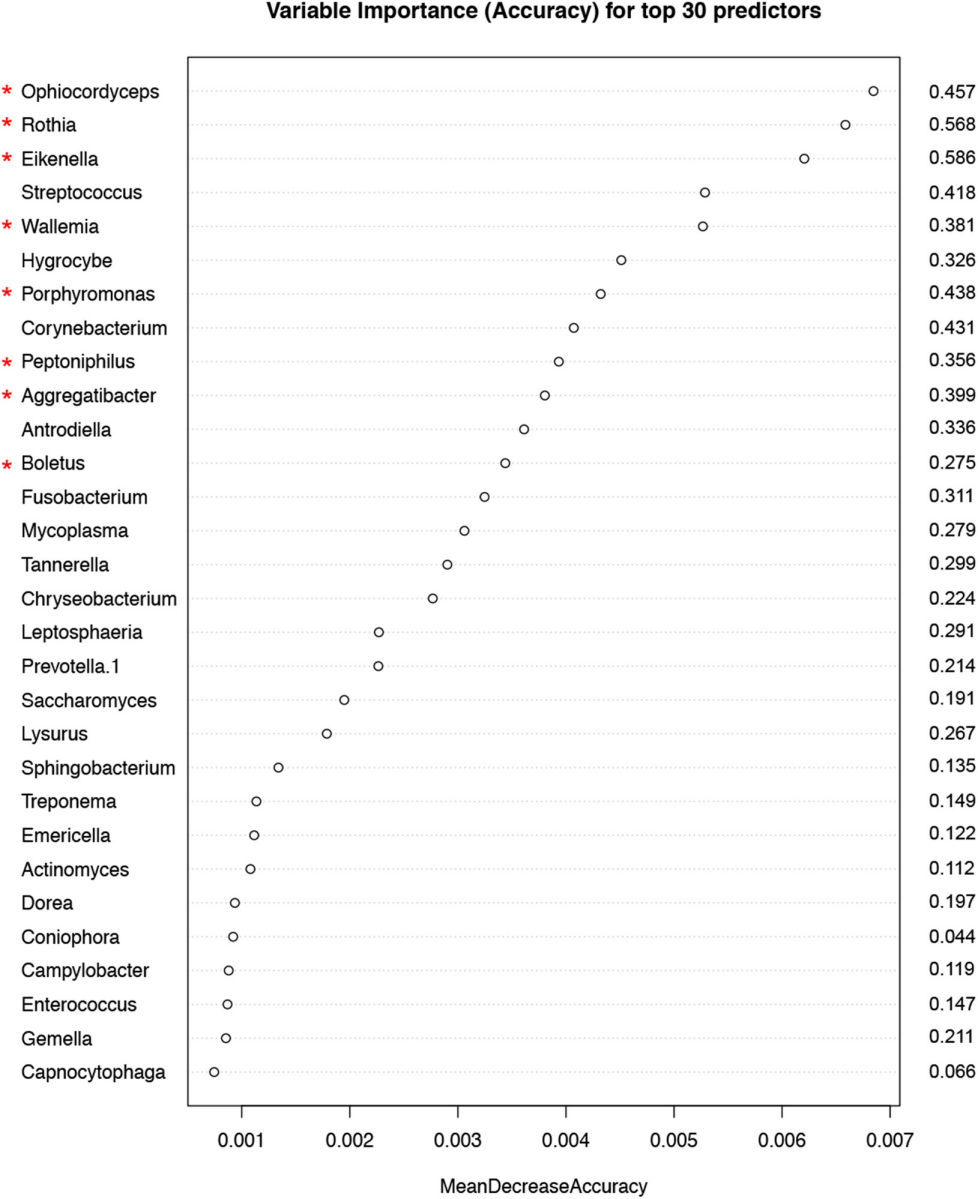
Variable importance measured by mean decrease in accuracy of top 30 predictor genera in the Random Forest (RF) model Numbers to the right of the plot indicate the proportion of bootstrapped samples (out of 1000) that the variable featured in after backwards variable selection using varSelRF. The red asterixes indicate the 8 variables contributing to the model with the optimum out-of-bag error rate (12%).

**Figure 6:**
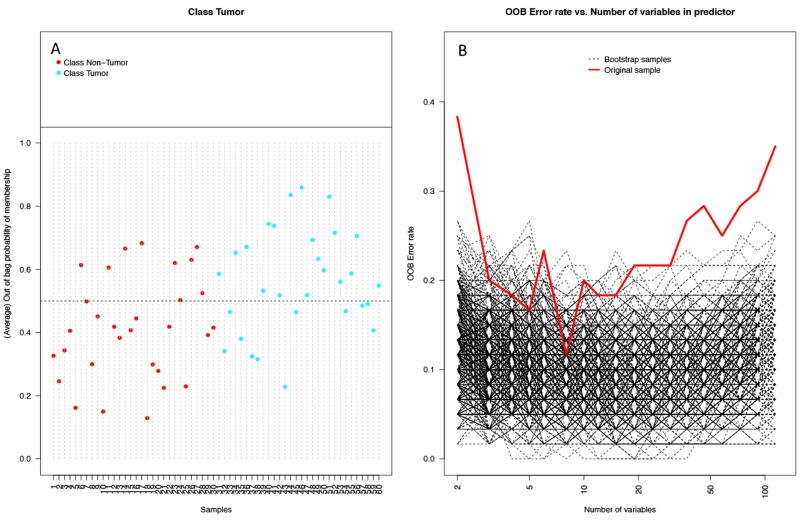
Performance of random forest (RF) model on 1000 bootstrap samples **(A)** Plot of each sample and its average out-of-bag (OOB) vote fraction. Non-tumor samples (red) above the dashed line and tumor samples (blue) below the dashed line were predicted incorrectly (9 non-tumor, 11 tumor), leading to an estimated error rate of 0.33 using the 0.632+ bootstrap method. **(B)** Plot of OOB error rates by number of variables used in the model in 1000 bootstrapped iterations of backwards variable selection using varSelRF. Both the bootstrap samples (black dashed lines) and original sample (red line) show OOB error rates dip at around 8 variables.

Random forest (RF) modeling based on both bacteriome and mycobiome showed that the relative abundances of 112 bacterial and fungal genera could distinguish tumor from non-tumor tissue with an accuracy of 70% (Figure 4A, 4B). In this model, the top 30 variables had higher predictive value for the groupings of tumor versus non-tumor as measured by mean decrease in accuracy than would be expected for a model in which samples were assigned to random groups (Figure 4C). Of the 15 bacterial genera found to be significantly different between tumor and non-tumor samples by Kruskal-Wallis analysis, 10 (*Rothia, Eikenella, Streptococcus, Porphyromonas, Aggregatibacter, Fusobacterium, Prevotella, Actinomyces, Campylobacter, Capnocytophaga*) were found among the RF model’s top 30 variables. Of the 3 fungal genera identified as significant by Kruskal-Wallis, only one (*Emericella*) was among the RF model’s top 30 variables. The variable importance was not very stable, with the majority of variables appearing in fewer than half of the backward variable selection iterations (Figure 5). The model’s OOB error rate of 30% was relatively stable upon evaluation using 1000 bootstrap iterations, with a 0.632+ estimate of prediction error at 33% (Figure 6A). Using backward variable selection on bootstrapped samples, the optimal number of variables was shown to be 8 (asterisks, Figure 5). An RF model constructed on the full data set using these 8 variables had an OOB error rate of 12%.

**Figure 7:**
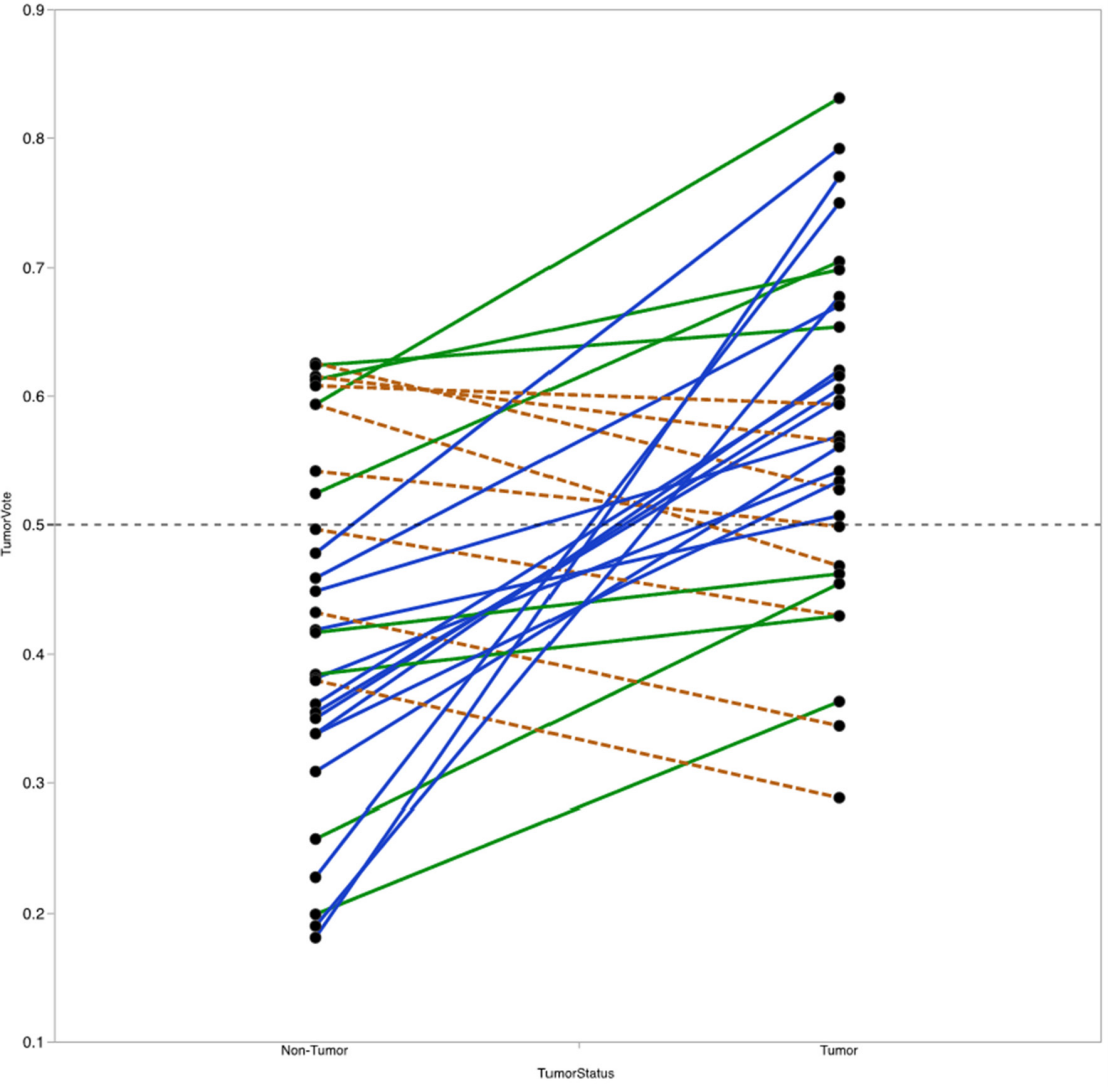
Vote fraction between matched tumor and non-tumor samples Lines connect the vote fractions of tumor (right) and non-tumor (left) samples. Samples connected by a solid blue line were predicted correctly by the Random Forest (RF) model; samples connected by a solid green or dashed orange line were predicted incorrectly by the RF model. Among incorrectly predicted samples, line color and type indicates directionality of the vote fraction difference (solid green line for decreased in non-tumor relative to tumor, dashed orange line for increased in non-tumor relative to tumor). Of the 14 with one incorrectly predicted sample, 8 had vote fraction differences that trended in the correct direction.

For each sample, the RF model reports the percentage of trees the sample traversed and was categorized as belonging to the tumor group, referred to here as the vote fraction. A vote fraction above 0.5 indicates the model predicted the sample to be tumor; a vote fraction below 0.5 indicates the sample is predicted to be non-tumor. The higher the vote fraction the more “tumor-like” the sample is, according to the model. We found that half of the patients with at least one incorrectly predicted sample still had tumor/non-tumor pairs with vote fractions that trended in the correct direction (Figure 7). In other words, the difference between vote fractions for the patient’s tumor sample and non-tumor sample was positive.

Next, we investigated whether the value of the difference in vote fractions for each individual patient correlate with any clinical features. We found that patients with high T-stage disease had lower mean differences between tumor and non-tumor samples compared with patients with low T-stage disease (mean difference 0.07 vs. 0.21, *P*=0.04). No other clinical features from Table 1 were significantly correlated with vote fraction differences.

**Figure 8:**
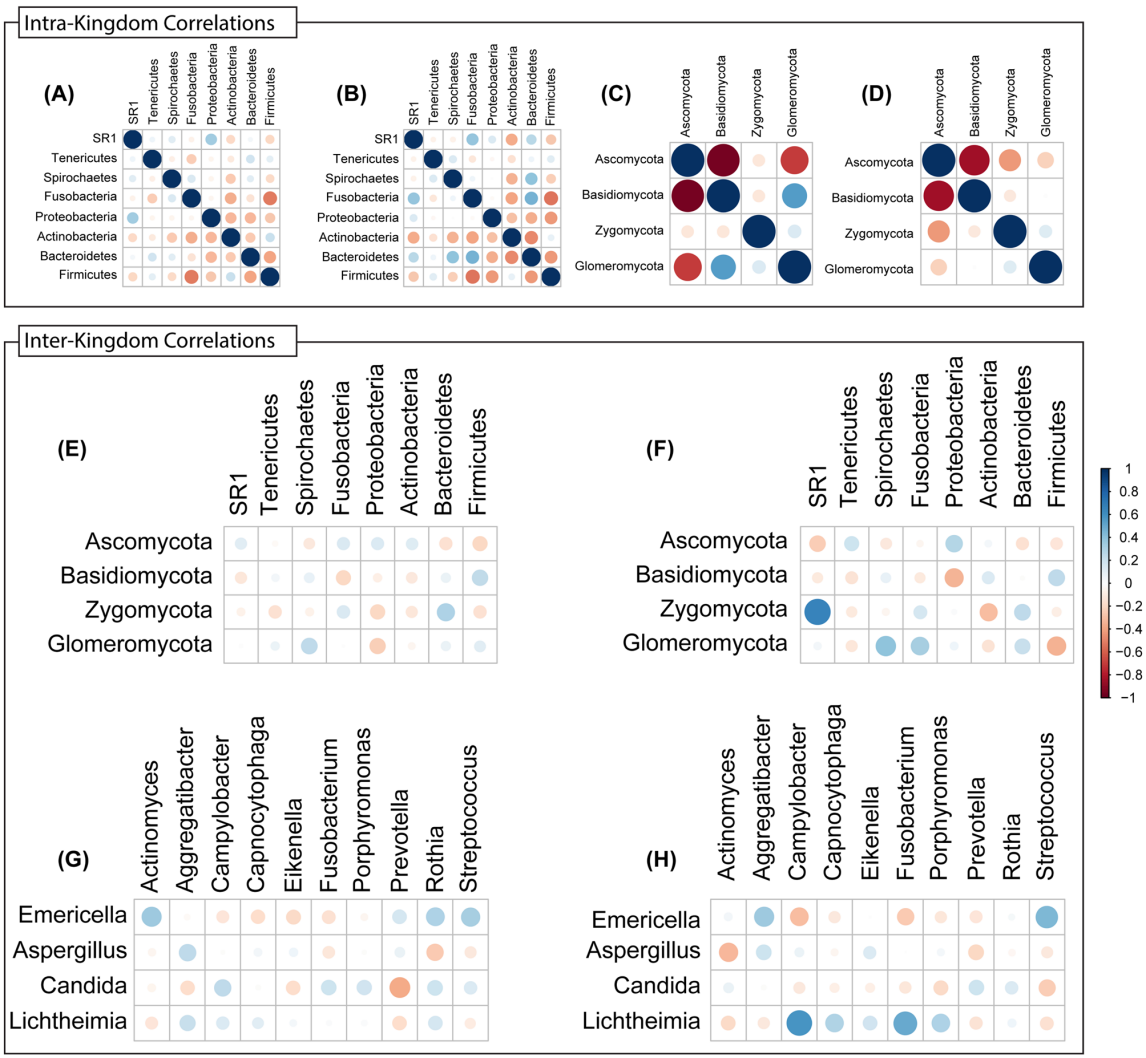
Intra- and Inter-Kingdom correlations at phylum and genus levels Intra-kingdom correlations are shown among bacterial phyla in **(A)** non-tumor or **(B)** tumor samples, and among fungal phyla in **(C)** non-tumor or **(D)** tumor samples. Inter-kingdom correlations observed between bacterial and fungal phyla in **(E)** non-tumor and **(F)** tumor samples, and at genus level in **(G)** non-tumor and **(H)** tumor samples. Blue – positive correlation, red – negative correlation. Circle size and color shading indicate value of correlation coefficient, with bigger circles with darker coloring representing higher coefficient values (maximum = 1) and smaller circles with lighter coloring representing lower coefficient values (minimum = 0).

Next, we conducted inter-and intra-kingdom correlation analyses with bacterial and fungal taxa in the microbiota of tumor and non-tumor samples. We found that the bacterial phylum Bacteroidetes exhibited robust positive intra-kingdom correlations with Fusobacteria and Spirochaetes in tumor samples (Figure 8A, 8B). At the fungal phylum level, negative correlation between Zygomycota and Ascomycota were increased while that between Glomeromycota and Ascomycota decreased in tumor samples (Figure 8C, 8D). Zygomycota also exhibited positive inter-kingdom correlation with two bacterial phyla (Fusobacteria and Bacteroidetes) and negative correlation with Actinobacteria (Figure 8E, 8F). At the genus levels, *Lichtheimia* correlated positively with *Campylobacter*, *Porphyromonas* and *Fusobacterium*, and negatively with *Actinomyces* (Figure 8G, 8H). Fungal species *Lichtheimia corymbifera* correlated positively with 11 bacterial species and negatively with 39 bacterial species (including *Lactobacillus* spp. Supplementary Table 3). These results show that specific inter- and intra-kingdom correlations exist within the bacterial and fungal microbiota in the setting of oral tongue cancer.

## DISCUSSION

Squamous cell carcinomas of the head and neck region are increasing in incidence globally. This trend is particularly exaggerated for mobile tongue cancers. Despite multidisciplinary research and advances in diagnosis and treatment, mortality rates have not significantly decreased over the last quarter century. While HPV has been identified as an etiologic agent in certain sites, it is rare in mobile tongue cancers, which still require exploration of etiology. Here, we show for the first time, bacteriome and mycobiome differences between mobile tongue cancers and their matched normal oral epithelium. Among the carcinomas, there also were differences in microbiome based on T stage. While our study and resulting data are novel, we acknowledge that our sample size is small, though counter-balanced by the relative homogeneity of the tumor site and HPV status (mainly negative).

The number of bacterial sequencing reads in our study were similar to other studies conducted with oral samples [17]. We found that the number of fungal sequence reads were lower than that of bacterial sequence reads, which also agrees with published studies showing that the number of fungal sequences are usually lower than that of bacterial sequences, with only ∼0.1% of detectable sequences in the total gut microbiota attributed to fungi and 99% to bacteria [18, 19]. Detection of lower number of fungal reads in a sample has been attributed to the fact that fungal signatures are more sensitive to DNA isolation method than bacterial signature [20] and limitations of the fungal sequencing databases used for identity assignments [21, 22]. However, in spite of these limitations, our analysis provides valuable insight into the potential clinical relevance of the bacteriome/mycobiome in oral tongue cancer.

The sample-wise variation in the tumor and non-tumor samples were similar, as revealed by PCO analysis. These distribution profiles are similar to those reported for bacteriome analyses of cancer samples [19, 23]. We also found that bacterial diversity and richness decreased in tumor samples, which is in agreement with the concept that reduction in microbial diversity is linked with disease in cancer [19, 24, 25]. Although microbiomes (bacteriomes) of specific body sites vary among individuals [26], the concept of a “core microbiome,” or shared bacteriome among individuals of the same body site, still exists [27]. Perturbations from the core bacteriome, or dysbiosis, have been seen in non-physiologic states, such as obesity. Although diversity index of mycobiome did not change between tumor and non-tumor samples, richness of the mycobiome was significantly reduced in tumor samples. Thus, our study suggests there is a change in the local environment with oral tongue cancer, manifested as distinct microbial dysbiosis, at both the bacterial and fungal levels. Dysbiosis could lead to an altered representation of bacterial genes and their metabolic pathways. This increased variation of microflora also could lead to changes in the abundance of certain species that promote tumorigenesis, the so-called “oncogenic bacteria” [28, 29].

To address our hypothesis that there is interplay between oropharyngeal bacteriome and mycobiome, we focused our RF modeling of both combined. As such, no one bacterial or fungal genus predominated in distinguishing the cancers from their normal tissues. Indeed, our RF model identified 10 bacterial genera (*Rothia, Eikenella, Streptococcus, Porphyromonas, Aggregatibacter, Fusobacterium, Prevotella, Actinomyces, Campylobacter, Capnocytophaga*), and only one fungal genus (*Emericella*) among the model’s top 30 variables. This is not surprising given the complexity of carcinogenesis. As examples, there are many genomic and epigenomic loci altered in head and neck carcinomas representing the multi-hit model of cancer. Here we show a multi-hit model of bacteria and fungi. We also show that the variable importance was not very stable which means that there is no obvious single genus driving the divide between tumor and not-tumor. It is important to note that this observation has no implication on how good our RF model is. The latter (goodness of the model) is measured by the out of bag error rate, which is, in fact, very stable, 30-33% with 1000 bootstraps. There are a few hypotheses that bear on biological significance in the context of lack of variable stability. One potential interpretation could be co-linearity of variables. It is unlikely that *Rothia* or *Eikenella* (as examples) alone drives tumorigenicity. Instead, if, e.g., *Rothia* and *Eikenella* trend together, and if *Rothia* is randomly selected as part of a model, then *Eikenella* may not be selected because it would not provide any additional discriminatory power. Thus, we may see *Rothia* included in half of the models and *Eikenella* the other half, just by chance. An alternate interpretation, which is more biologically attractive, would be based on the thesis that there are multiple community structures (bacterial/fungal “hits”) that contribute to the same “phenotype” (outcome), i.e., tumor versus not-tumor. As an example, *Rothia*-high and *Fusobacterium*-low predicts for tumor but *Eikenella*-high and *Fusobacterium*-high also is associated with tumor, but perhaps via a different mechanism.

Our correlation analyses revealed interesting associations between microbes and tumor status, which could be detected at phylum as well as genus level analyses. In this regard, phylum level correlations indicated a positive association between Zygomycota (which includes the genus *Lichtheimia*) and Bacteroidetes (which includes *Porphyromonas*) and Fusobacteria (includes *Fusobacterium*). Similarly, the negative correlation between Lichtheimia and Actinomyces was predicted by the association between their respective phyla (Zygomycota vs. Actinobacteria). These analyses also hinted at interesting inter-kingdom interplay. For example, the Gram-negative bacteria *Campylobacter*, *Fusobacterium* and *Porphyromonas* were negatively associated with *Emericella*, but positively with *Lichtheimia*. The fact that tumor samples exhibited decrease in abundance of *Emericella* could suggest that the corresponding increase in *Campylobacter*, *Fusobacterium* and *Porphyromonas* may be happening concomitantly as the levels of *Lichtheimia* (the fungus *Mucor*) are increasing. *Lichtheimia* is associated with infections in immunocompromised (including cancer) patients [30-33], while the bacterial genera *Fusobacterium* and *Porphyromonas* (known periodontal pathogens) and *Campylobacter* (common in GI infections) are major constituents of the “mobile microbiome” originating in the oral cavity but also associated with extra-oral infections and inflammation [34]. The correlations observed in the current study with oral tongue cancer patients may indicate that microbial dysbiosis reflects changes in the immune status due to the underlying disease. For example, increasing *Lichtheimia* levels may be linked to attenuated immunity, while increasing levels of oral pathogens like *Fusobacterium* and *Porphyromonas* may be due to changes in the mucosal surface. The link between these microbiota level changes and host response may indeed be induced by therapy, and/or may be predictive for the disease. The cause-versus-effect relationships between these organisms should be investigated.

Another corollary to the changes in microbiota is the potential effect of metabolites secreted by these organisms on cancer. Such a concept has been investigated by other investigators also, who previously identified several “oncogenic bacteria”. In this regard, acetaldehyde (derived from alcohol metabolism) has been proposed as the oral carcinogen linked to oral cancers due to alcohol consumption. In the presence of alcohol exposure and increased abundances of microbes that produce acetaldehyde, such as *Rothia, Streptococus* and *Prevotella* [35], we could postulate that increased oral acetaldehyde could promote oral carcinogenesis. The fact that fungal abundance is also significantly altered between the tumor and non-tumor groups, and since fungi (e.g. *Candida albicans* [36, 37] are known to mediate production of salivary acetaldehyde in patients with ethanol-associated oral cancer, these fungi may represent “oncogenic fungi.” Further studies are warranted to investigate the oncogenic potential of the fungal species *Lichtheimia corymbifera*.

Correlation analyses also showed that *Lichtheimia corymbifera* was negatively correlated with *Lactobacillus* spp., which could be related to changes in the local gut environment that favors increased abundance of specific taxa. *Lactobacillus* are generally regarded as beneficial bacteria that regulate the growth of bacteria and fungi [38, 39, 40]. Reduction in levels of *Lactobacillus* spp. may induce changes in the microbial ecosystem of oral tongue cancer patients, which in turn can influence the conditions (e.g. pH, micronutrients) for microbial dysbiosis.

It is notable that *Emericella* is decreased in abundance in cancer tissues compared to their normal tissue. *In vitro* studies show *Emericella* exposure resulted in increased p53 tumor suppressor expression, at least in colon cancer cells [41]. Thus, the observed decrease in abundance of this genus in tumor samples may have led to a decrease in expression of the p53 tumor suppressor, thus contributing to the oral tongue cancer phenotype. A potential, although provocative, outcome of such association could be that *Emericella* may have utility as a probiotic in managing oral tongue cancer.

Of all the clinico-pathologic features, we only found an association of T stage with bacteriome-mycobiome profile differences. Those with high T-stage had lower mean differences between their tumors and adjacent normal tissue. In contrast, patients with low T-stage had a larger mean difference between their tumors and matched normal tissue. Interestingly, Spirochaetes was already relatively abundant in the non-tumor normal oral tissue in those with high T stage compared to the normal oral tissue from those with low T stage in the context that Tenericutes, Spirochaetes, and Bacteroidetes are over-represented in the tumors of those with high T stage. These observations could suggest that there already exist bacteriomic/mycobiomic dysbioses in the normal (appearing) tissue in high T-stage tumors. It has long been believed that a field cancerization effect is important in the pathogenesis of head and neck squamous cell carcinomas. Typically, this field effect is shown by similar somatic genetic and epigenetic/transcript expression differences between the oral carcinomas and their matched non-tumor oral tissue [42]. Interestingly, over-expression of Ki67 (a marker of rapidly dividing cells) in normal oral epithelium distant from oral cancers has been shown to have a poor prognosis [43]. Here, our observations are consistent with a bacteriome/mycobiome field effect where the presence of dysbiosis in the normal tissues associates with larger tumors. Based on our observations, some may cautiously speculate that elimination of bacterial and fungal dysbioses may prevent or slow disease progression. Our pilot data should be confirmed in larger series and the speculation of rebalancing bacterial/fungal dysbioses should be directly addressed, perhaps in non-human animal models.

## MATERIALS AND METHODS

### Patient sample collection and demographics

This study was approved by the Cleveland Clinic Institutional Review Board for Human Subjects Protection. Written informed consent was obtained from all cases. Fifty-three unrelated patients with mobile tongue cancer undergoing resection were prospectively enrolled (2003-2014). Of these 53, 40 had adequate fresh-frozen specimens (30-50mg) of matched tumor and adjacent normal tissues and were collected into a tissue biorepository under a protocol specifically designed to maintain sterility for biomic analyses. Matched normal tissues were resected approximately 2cm from the tumor margin, were aseptically collected in the operating room, flash frozen, and stored at -80°C. Relevant clinicopathologic features at the time of diagnosis were collected, with summary demographics shown in Table 1. Of the 40-matched normal-tumor sets, 39 had sufficient tissue for both bacteriome and mycobiome analyses. Two of the 39 tumor samples failed at amplification step and did not yield any sequencing data for bacteriome analysis and were removed from analyses.

### DNA extraction

Total DNA was extracted using a previously described protocol [9] with modifications as follows. Unlike extraction of DNA from bacterial cells, extracting DNA from fungal cells is more challenging and requires optimization. It is well established that methods for genomic DNA extraction from fungal cells require hours to days to complete and often incorporate toxic chemicals. Additionally, the release of DNA is often poor due to cell walls or capsules that are not readily susceptible to lysis. To optimize DNA extraction from clinical samples, we conducted preliminary experiments using *Aspergillus fumigatus* as a representative fungus known to pose a challenge to lysing and identified the optimal setting (6 m/s, 3 runs of 60s) for DNA extraction of fungal DNA. This setting was also optimal for DNA extraction form bacterial cells. Microbial (fungal and bacterial) genomic DNA was isolated and purified with the QiaAmp DNA Stool mini Kit (Qiagen) following the manufacturer’s instructions with minor modifications. Briefly, 3 additional bead-beating steps (Sigma-Aldrich beads, diameter = 500 μm) with the MP Fastprep-24 (speed setting of 6, 3 runs of 60s), after the stool lysis step (in ASL buffer) were performed. The quality and purity of the isolated genomic DNA was confirmed spectrophotometrically using NanoDrop 2000 device (Fisher Scientific SAS, Illkirch, France). DNA concentration was quantified using the Qubit 2.0 instrument applying the Qubit dsDNA HS Assay (Life Technologies, USA). Extracted DNA samples were stored at -20°C.

### Microbiome analyses

Analysis of the mycobiome profile in the extracted DNA samples was conducted as described previously by our group [44, 45]. A brief summary of the method is provided below.

#### Amplicon library preparation

The Internal Transcribed Spacer 1 (ITS1) and 16S rDNA regions for fungi and bacteria, respectively, were amplified as described previously [45]. Briefly, ITS1 region was amplified using ITS1F (CTTGGTCATTTAGAGGAAGTAA) and ITS 2 (GCTGCGTTCTTCATCGATGC) primers. The reactions were carried out on 100 ng template DNA, in 50 μl (final volume) reaction mixture consisting of Dream Taq Green PCR Master Mix (Thermo Scientific), 0.1g/L bovine serum albumin, 1% of dimethylsulfoxide (DMSO), 6 mM MgCl_2_, and a final primer concentration of 400nM. Initial denaturation at 94°C for 3 min was followed by 35 cycles of denaturation for 30s each at 94°C, annealing at 50°C for 30 s, and extension at 72°C for 1 min. Following the 35 cycles there was a final extension time of 5 min at 72°C.

The V4 region of the 16S rRNA gene was amplified using16S-515F: GTGCCAGCMGCCGCGGTAA and 16s-806R: GGACTACHVGGGTWTCTAAT primers. The reactions were carried out on 100 ng template DNA, in 50 μl (final volume) reaction mixture consisting of Dream Taq Green PCR Master Mix (Thermoscientific), 0.1g/L bovine serum albumin, 1% of dimethylsulfoxide (DMSO), 6 mM MgCl_2_, and a final primer concentration of 400nM. Initial denaturation at 94°C for 3 min was followed by 30 cycles of denaturation for 30 s each at 94°C, annealing at 50°C for 30 s, and extension at 72°C for 1 min. Following the 30 cycles there was a final extension time of 5 min at 72°C. The size and quality of amplicons was screened by 1.5% Tris Acetate EDTA agarose gel electrophoresis, using 100v and electrophoresed for 45 min and stained with ethidium bromide.

The PCR products were sheared for 20 min, using Ion Shear Plus Fragment Library Kit (LifeTechnologies, NY, USA). The Amplicon library was generated with sheared PCR products using Ion plus Fragment Library kits (<350 bp) according to the manufacturer’s instructions. The library was barcoded with Ion Xpress™ Barcode Adapter, and ligated with the A and P1 adaptors.

#### Sequencing, classification and analysis

The adapted barcoded libraries were equalized using the Ion Library Equalizer kit to a final concentration of 100 pM. Once equalized, the samples were pooled and diluted to 26 pM, and attached to the surface of Ion Sphere particles (ISPs) using an Ion PGM Template OT2 200bp kit v2 (LifeTechnologies, USA) according to the manufacturer’s instructions, via emulsion PCR. Quality of ISPs templates was checked using Ion Sphere™ Quality Control Kit (Part no. 4468656) with the Qubit 2.0 device. Sequencing of the pooled libraries was carried out on the Ion Torrent Personal Genome Machine (PGM) system using the Ion Sequencing 400 bp kit (all from LifeTechnologies) for 150 cycles (600 flows), with a 318 chip following the manufacturer’s instructions. De-multiplexing and classification was performed using the Qiime 1.6 platform. The resulting sequence data were trimmed to remove adapters, barcodes and primers during the de-multiplexing process. In addition, the bioinformatics process filters were applied to the sequence data for the removal of low-quality reads below Q25 Phred score and denoised to exclude sequences with read length below 100 bp [46]. De novo operational taxonomic units (OTUs) were clustered using Uclust algorithm and defined by 97% sequence similarity [47]. Classification at the species level was referenced using the UNITE 5.8s database and taxa assigned using the nBlast method with a 90% confidence cut-off [48, 49]. Chimeras where removed during noise removal post assigning taxonomy due to the low representation (below 0.01%).

### Bioinformatics and statistical analyses

The statistical programming language R (version 3.3.1) [50] and related packages [51] were used for diversity and correlation analyses, and Kruskal-Wallis (non-parametric) analysis of variance using abundance data. Euclidean distance were calculated using the *dist* function (base *R*) and vegan::vegdist [52-55]. *hist* function was used to create a histogram to visualize distribution of distance dissimilarities. UniFrac Distances were calculated using the *vegan* and *GUniFrac* packages. The *hclust* function (base R) was used to conduct hierachical clustering in dendograms to visualize distance between groups. Principle coordinate analysis (PCoA was conducted using the *cmdscale* function (base R).

Diversity was analyzed in an unbiased manner using Shannon diversity index (characterizes species diversity) and richness (number of organisms in a sample) at all taxonomical levels using the R package ‘*vegan*’ [52]. Abundance data are presented as proportions (relative abundance) within each analyzed sample group. Correlation analyses were performed using the R package ‘*corrplot*’ [56]. All group-wise comparisons were conducted with base statistics functions using the Pairwise Multiple Comparison of Mean Ranks (PMCMR) package in R [57], employing Kruskal & Wallis test followed by Bonferroni-Dunn post-hoc adjustment. *P* <.05 was considered statistically significant for all tests (after correcting for multiple comparisons).

Random forest (RF), an ensemble learning method based on classification trees, was implemented using the R package ‘*randomForest*’, with the relative abundances of 112 bacterial and fungal genera used as variables to predict tumor or non-tumor status [58, 59]. RF grows a specified number (ntree=2001) of classification trees on a specified number (mtry=10) of randomly selected input variables at each node. Each tree is constructed on a bootstrapped sample from the original data set that constitutes approximately two thirds of the samples. The one third of samples not used to construct the tree is then used to evaluate the accuracy of the tree, thus making RF models relatively robust against overfitting [58]. The aggregate error rate from the entire forest is reported as the out-of-bag (OOB) error rate, and variables can be ranked by importance based on the number of times they are used as splitters. RF has been shown to outperform discriminant analysis and support vector machines in microarray analysis and microbiome analyses [60, 61]. Backwards iterative variable selection and evaluation of the stability of the model (OOB error rate and variable importances) was performed using 1000 bootstrapped samples through R package ‘*varSelRF*’ with default settings except c.sd set to 0 [62]. Nine tumor/non-tumor pairs were excluded from the analysis due to insufficient bacterial sequence counts (<50 reads). Analysis across groups on vote fraction differences between matched tumor and non-tumor pairs was done using MANOVA with repeated measures using JMP Pro 12 (SAS Institute Inc., Cary, NC).

## SUPPLEMENTARY MATERIALS FIGURES AND TABLES


