# Supplementary material for: Bacteriome and mycobiome associations in oral tongue cancer
Source: Oncotarget. 2017 Oct 19;8(57):97273–89. doi: 10.18632/oncotarget.21921 (PMC5722561; doi:10.18632/oncotarget.21921)
Supplement: Supplementary file 1 [file oncotarget-08-97273-s001.pdf]

Bacteriome and mycobiome associations in oral tongue cancer

SUPPLEMENTARY MATERIALS

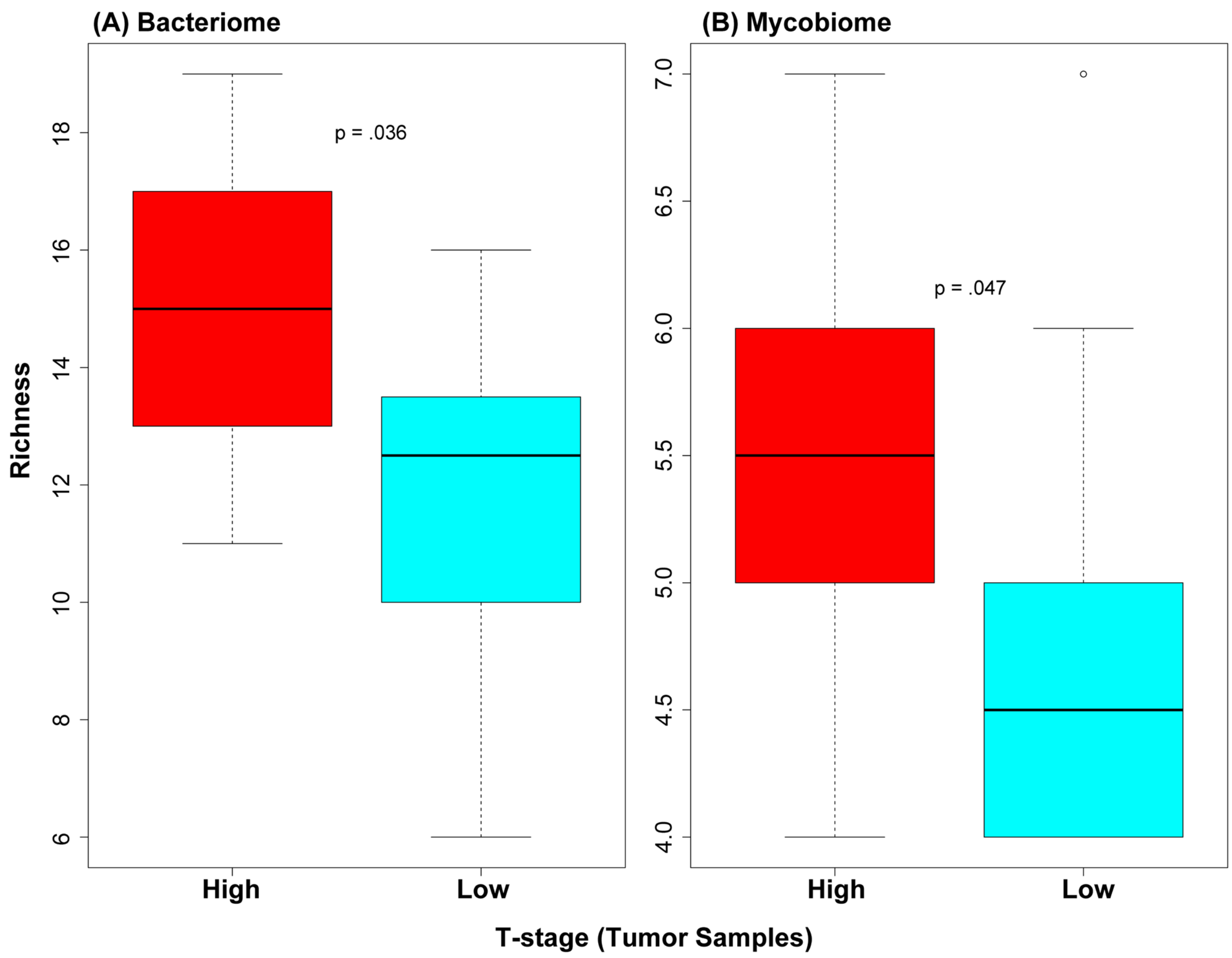

Supplementary Figure 1: Box and whisker plots of richness of **(A)** bacterial phyla and **(B)** fungal phyla in tumor samples with high and low T-stage. Description of data: Dark horizontal lines represent the median, with the box representing the first (Q1) and third (Q3) quartiles, the outer fences representing 1.5 x interquartile range, and the black circles representing outliers. *P* <.05 for all comparisons between T-stages.

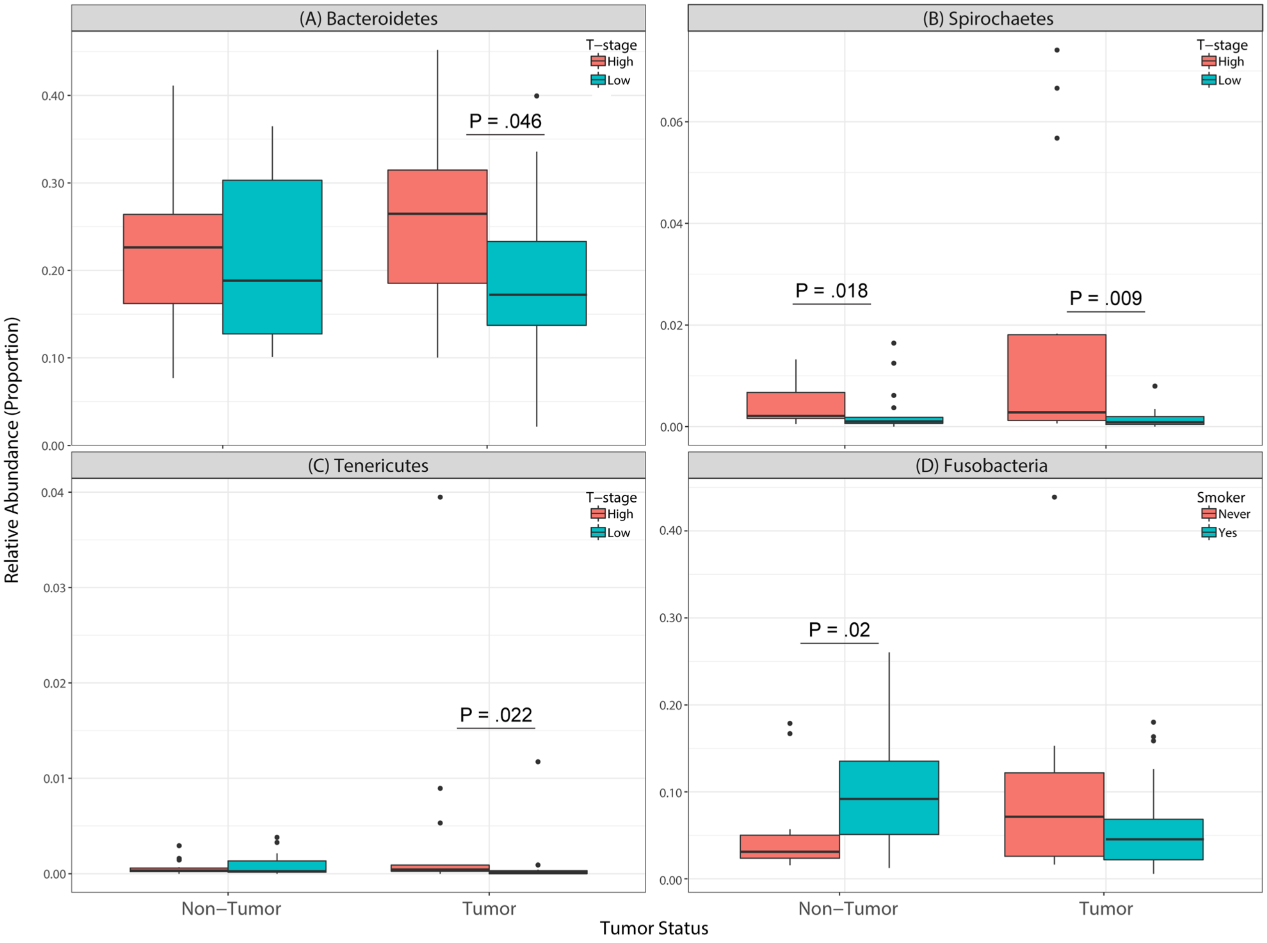

Supplementary Figure 2: Box and whisker plots of relative abundance of four bacterial phyla found significantly different in oral tongue tumor samples and their matched normal tissue samples, between different T-stages or smoking status. Description of data: Data were compared between **(A-C)** T-stage or **(D)** smoking status in the non-tumor and tumor groups. Dark horizontal lines represent the median, with the box representing the first (Q1) and third (Q3) quartiles, the outer fences representing 1.5 x interquartile range, and the black circles representing outliers. *P* <.05 for all comparisons between T-stages or smoker groups.

Supplementary Table 1 : Relative abundance of bacterial phyla in non-tumor and tumor groups. Description of data: Relative abundance is expressed as mean percent composition +/- standard deviation (SD). P-values reflect comparison between the tumor samples and non-tumor matched normal oral samples

Genus	Non-Tumor		Tumor		p-value
	Mean	SD	Mean	SD	
Streptococcus	22.4%	9.8%	34.2%	12.1%	0.0002
Prevotella	19.8%	10.0%	14.2%	7.7%	0.0262
Fusobacterium	7.7%	8.9%	2.8%	3.1%	0.0003
Actinomyces	4.2%	4.1%	8.1%	6.0%	0.0021
Rothia	3.8%	2.9%	8.4%	5.9%	0.0003
Capnocytophaga	3.2%	4.4%	1.6%	2.7%	0.0320
Parvimonas	3.0%	3.1%	1.5%	2.3%	0.0043
Gemella	2.9%	2.2%	1.9%	1.5%	0.0455
Porphyromonas	2.6%	3.8%	1.1%	2.4%	0.0004
Campylobacter	2.3%	3.5%	1.3%	2.1%	0.0253
Peptostreptococcus	1.7%	2.4%	0.7%	1.1%	0.0070
Aggregatibacter	1.6%	3.6%	0.3%	0.3%	0.0013
Treponema	0.8%	1.6%	0.5%	1.4%	0.0262
Eikenella	0.7%	1.6%	0.2%	0.2%	0.0002
Peptoniphilus	0.5%	1.0%	0.3%	0.8%	0.0024
Mycoplasma	0.3%	0.7%	0.0%	0.1%	0.0017
Tannerella	0.2%	0.6%	0.2%	0.4%	0.0135
Corynebacterium	0.2%	0.2%	0.3%	0.4%	0.0017
Enterococcus	0.2%	0.1%	0.3%	0.5%	0.0056
Micrococcus	0.1%	0.1%	0.2%	0.3%	0.0166
Desulfobulbus	0.1%	0.3%	0.0%	0.0%	0.0328
Sphingobacterium	0.0%	0.2%	0.0%	0.0%	0.0083

Supplementary Table 2 : Relative abundance of fungal phyla in non-tumor and tumor groups. Description of data: Relative abundance is expressed as mean percent composition +/- standard deviation (SD). P-values reflect comparison between the tumor samples and non-tumor matched normal oral samples

Fungal Genus	Non-Tumor		Tumor		p-value
	Mean	SD	Mean	SD	
Wallemia	1.2%	2.3%	1.2%	1.0%	0.047
Lysurus	0.6%	1.5%	0.2%	0.3%	0.010
Leptosphaeria	0.6%	0.7%	0.3%	0.4%	0.010
Antrodiella	0.4%	0.4%	0.2%	0.2%	0.005
Boletus	0.1%	0.2%	0.0%	0.1%	0.013
Ophiocordyceps	0.1%	0.1%	0.4%	0.9%	0.037
Guignardia	0.0%	0.1%	0.1%	0.2%	0.043

Supplementary Table 3 : Correlations of *Lichtheimia corymbifera* with bacterial species. Description of data: Positive correlations of bacterial species with *L. corymbifera* appear in the left columns and negative correlations on the right

Positive Correlations		Negative Correlations	
**Bacterial spp.**	**Coefficient**	**Bacterial spp.**	**Coefficient**
Shuttleworthia.satelles	0.435909214	*Staphylococcus.epidermidis*	-0.265917391
Capnocytophaga.ochracea	0.347096427	*Prevotella.intermedia*	-0.260238735
Atopobium.rimae	0.244680067	*Alcaligenes.faecalis*	-0.246697875
Micrococcus.luteus	0.181494183	*Enterococcus.casseliflavus*	-0.24528205
Prevotella.nigrescens	0.145370313	*Treponema.amylovorum*	-0.232148847
Sphingobacterium.multivorum	0.118410347	*Bifidobacterium.longum*	-0.223557894
Rothia.mucilaginosa	0.082514308	*Salinivibrio.costicola*	-0.218429299
Streptococcus.anginosus	0.059967363	*Bifidobacterium.adolescentis*	-0.198154189
Haemophilus.parainfluenzae	0.052960097	*Propionibacterium.acnes*	-0.193964683
Prevotella.copri	0.045408443	*Bacillus.cereus*	-0.18468058
Neisseria.subflava	0.002149111	*Uruburuella.suis*	-0.183393572
		Acinetobacter.guillouiae	-0.161631963
		Atopobium.vaginae	-0.160834994
		Lactobacillus.iners	-0.1556918
		Corynebacterium.kroppenstedtii	-0.152892791
		Lactobacillus.salivarius	-0.15263367
		Streptococcus.alactolyticus	-0.147046175
		Porphyromonas.endodontalis	-0.140476415
		Rothia.dentocariosa	-0.133812383
		Prevotella.melaninogenica	-0.133310433
		Bifidobacterium.pseudolongum	-0.116987349
		Prevotella.nanceiensis	-0.107137806
		Brevibacterium.aureum	-0.098438171
		Pseudomonas.veronii	-0.097688359
		Acinetobacter.johnsonii	-0.089697586
		Haemophilus.influenzae	-0.084159388
		Prevotella.pallens	-0.07238422
		Campylobacter.ureolyticus	-0.071050246
		Peptostreptococcus.anaerobius	-0.070198508
		Lactobacillus.vaginalis	-0.066489669
		Lactobacillus.reuteri	-0.058953699
		Neisseria.bacilliformis	-0.058065788
		Roseateles.depolymerans	-0.052908831
		Prevotella.tannerae	-0.049806743
		Bacillus.flexus	-0.044219818
		Aggregatibacter.segnis	-0.031707819
		Streptococcus.infantis	-0.028045957
		Rothia.aeria	-0.021624684
		Bergeriella.denitrificans	-0.004421025
